# Androgen Receptor Imaging in the Management of Hormone-Dependent Cancers with Emphasis on Prostate Cancer

**DOI:** 10.3390/ijms24098235

**Published:** 2023-05-04

**Authors:** Kalevi Kairemo, Marina Hodolic

**Affiliations:** 1 Department of Molecular Radiotherapy & Nuclear Medicine, Docrates Cancer Center, 00180 Helsinki, Finland; 2 Department of Nuclear Medicine, The University of Texas MD Anderson Cancer Center, Houston, TX 77030, USA; 3 Department of Nuclear Medicine, Faculty of Medicine and Dentistry, Palacký University, 779 00 Olomouc, Czech Republic; marina.hodolic@gmail.com

**Keywords:** FDHT, PET, androgen receptor, receptor targeting, fluorine radioisotopes, endocrine therapy, prostate cancer, breast cancer, glioma, therapy response

## Abstract

Prostate cancer is dependent on the action of steroid hormones on the receptors. Endocrine therapy inhibits hormone production or blocks the receptors, thus providing clinical benefit to many, but not all, oncological patients. It is difficult to predict which patient will benefit from endocrine therapy and which will not. Positron Emission Tomography (PET) imaging of androgen receptors (AR) may provide functional information on the likelihood of endocrine therapy response in individual patients. In this article, we review the utility of [^18^F]FDHT-PET imaging in prostate, breast, and other hormone-dependent cancers expressing AR. The methodologies, development, and new possibilities are discussed as well.

## 1. Introduction

**Figure 1:**
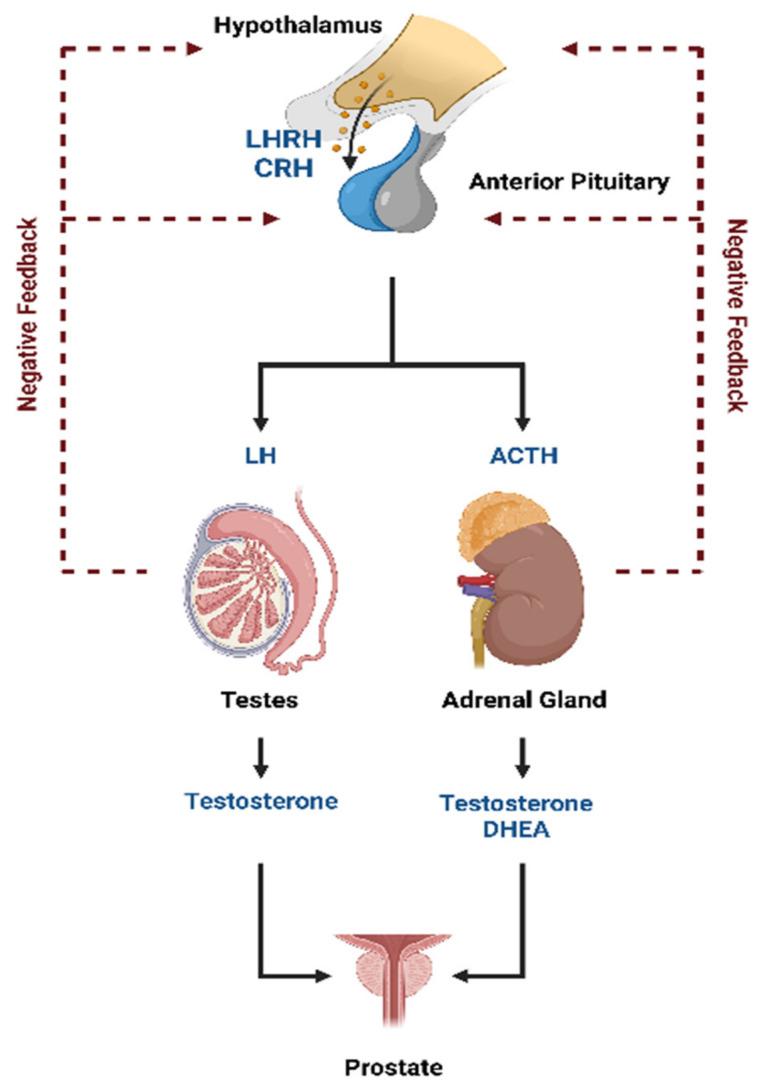
Hormonal regulation of androgen production. This figure is modified from [2]. Abbreviations: ACTH = adrenocorticotropic hormone, CRH = corticotropin-releasing hormone, DHEA = dehydroepiandrosterone, LH = luteinizing hormone, and LHRH = luteinizing hormone-releasing hormone.

Prostate cancer is a hormone-sensitive cancer. Tumor growth is dependent on androgens, such as testosterone and its derivative 5α-dihydrotestosterone (DHT). More than 90% of testosterone is synthesized in the testicles, while the remaining is mainly produced in the adrenal glands [1]. The luteinizing hormone-releasing hormone (LHRH) and the corticotropin-releasing hormone (CRH) produced by the hypothalamus stimulate the release of the luteinizing hormone (LH) and the adrenocorticotropic hormone (ACTH), respectively, from the anterior pituitary gland into the blood (Figure 1). The LH activates the production of testosterone caused by the Leydig cells in the testicles while ACTH triggers the production of testosterone and the other androgen dehydroepiandrosterone (DHEA) from the adrenal glands. A negative feedback loop regulates hypothalamic and pituitary hormone secretion.

**Figure 2:**
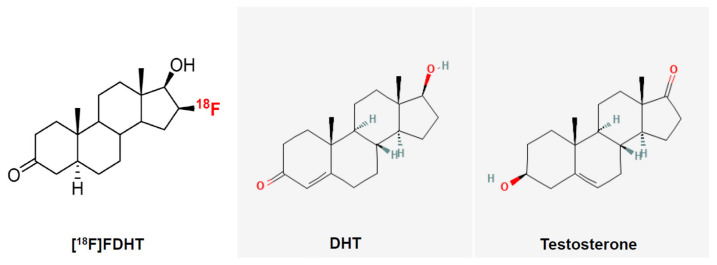
Chemical structures of 16β-[^18^F]fluoro-5α-dihydrotestosterone (FDHT), 5α-dihydrotestosterone (DHT), and testosterone. The PET tracer used in study is shown on white background, nonradioactive compounds on grey.

The activity of the androgens is mediated via the androgen receptor (AR), which is expressed both in the prostate epithelial cells and stromal cells [3]. The stroma of the prostate consists of cells (fibroblasts, smooth muscle cells, endothelial cells of capillaries, and lymphatic vessels) and the extracellular matrix. The epithelium of the prostate consists of secretory, neuroendocrine, intermediate, basal, and stem cells. There is a constant interaction: androgenic and estrogenic hormones stimulate stroma that further stimulates the growth, proliferation, and secretion of the epithelium [3]. Once inside the prostatic cells, testosterone is converted, by the enzyme 5-α-reductase, into the more potent AR ligand, DHT. Their chemical structures are shown in Figure 2. The AR binds the androgens in the cytoplasm and translocate into the nucleus where it, in complex with other coregulator proteins, induces or maintains the transcription of AR-targeted genes, including genes involved in cell growth, proliferation, and survival as well as genes encoding seminal proteins, such as the PSA [1,4]. Malignant prostate cells exhibit an excess activation of the androgen signaling pathway, resulting in an uncontrolled proliferation of tumor cells [4]. The increased PSA levels that can be detected in the serum of patients with prostate cancer are believed to result from disruptions of the prostatic cell and basement membranes [5].

Androgenic hormones, such as testosterone and DHT, regulate the development and maintenance of male sexual phenotype by binding to AR. The AR, also known as NR3C4 (nuclear receptor subfamily 3, group C, member 4), is a type of nuclear receptor. The AR is most closely related to the progesterone receptor; progestins in higher dosages can block the AR [6,7]. By activating the AR, specific gene expression is activated, and both normal and cancerous prostate cells grow. The inhibition of AR activity may delay prostate cancer progression. New therapies directed against the AR and AR signaling have shown a clear survival benefit in patients with prostate cancer [8,9]. As long as AR is present in prostate cancer, it typically remains an effective target for hormone-directed therapies. Even in castrate-resistant prostate cancer (CRPC), the AR plays a major role in tumor growth. In such conditions, the response to second- and third-line hormonal therapies is of short duration, and is associated with an overexpression of AR [10]. Chen et al. confirmed that AR overexpression is associated with resistance to bicalutamide [11]. Additionally, mutations and alterations in the relative expression of AR may contribute to the progression of prostate cancer and the failure of endocrine therapy. For example, the conversion from CRPC to neuroendocrine-type prostate cancer usually leads to the loss of AR and responsiveness to AR-targeted therapies [12].

Fluoro-5α-dihydrotestosterone (FDHT) is a radiolabeled androgen agonist that was developed for clinical use. The chemical structures of FDHT, DHT, and testosterone are shown in Figure 2.

First-in-man/woman steroid hormone receptor targeting studies were reported for 16α-[^18^F]fluoroestradiol (FES) in 1984 [13], 16β-[^18^F]-fluoro-5-dihydrotestosterone ([^18^F]FDHT) in 2005 [14], and 21-[18F]-fluoro-furanyl-nor-progesterone (FFNP) in 2012 [15].

Initial studies from Memorial Sloan–Kettering Cancer Center (MSKCC) demonstrated the feasibility, in vivo targeting, and biokinetics of 16β-^18^F-fluoro-5α-dihydrotestosterone (FDHT) PET in patients with metastatic prostate cancer. The metabolism of FDHT turned out to be rapid, with 80% conversion within 10 min to radiolabeled metabolites that circulated while bound to plasma proteins. The tumor uptake was also rapid and tumor retention was prolonged.

In our review article, we focus on FDHT in the AR targeting of prostate, breast, and other cancers.

## 2. [^18^F]FDHT PET in Patients with Prostate Cancer

### 2.1. Developmental History of Androgen Receptor Imaging

The earliest reports of AR imaging studied radiobrominated [^77^Br] and radioiodinated [^125^I] androgens in prostate cancer. The AR uptake of both tracers was poor, and both tracers were chemically or metabolically unstable [16,17,18,19]. More recent work on radioiodinated androgens showed that 7α-iodo or 17α-iodovinyl groups bound well to the receptor, but the in vivo biodistribution studies were unpromising [20,21]. The binding affinity of the major circulating androgen, testosterone, to the AR was not high when compared to the subnanomolar affinity of estradiol for the ER (K_d_ = 0.2 nM). The affinity of testosterone for AR was 10-fold lower (K_d_ = 2 nM), whereas DHT binds 10-fold better (K_d_ = 0.2 nM). The development of an imaging agent was performed by Katzenellenbogen’s group [22]. They initiated from the testosterone and used four high affinity ligands: 5α-dihydrotesterone (i.e., DHT), 19-nortestosterone, mibolerone, and methyltrienolone (this compound was developed by Roussel with a name, R1881). Additionally, the fluorine-labelled analogs were analyzed for their binding affinities. Methyltrienolone had a relative affinity of 100 and the three selected compounds for further study had relative affinities from 31 to 57 (Table 1). This was investigated by comparing the effect of reducing endogenous testosterone levels by castration vs. by feedback inhibition (mechanism in Figure 1) from pretreatment with high doses of an estrogen (diethylstilbesterol, DES) on the biodistribution of [^3^H]R1881, [^3^H]testosterone, [^3^H]19-nortestosterone, [^3^H]DHT, and [^3^H]mibolerone [22]. In all cases, they found AR-selective uptake in the prostate, and although castration more markedly reduced the AR occupancy by endogenous androgens, high-dose estrogen pretreatment was nearly as effective and much more convenient [23]. Scientists found that some radio fluorinated derivatives had rapid defluorination as shown by a bone uptake that was not reduced by a blockade of excess unlabeled DHT [24,25,26]. Defluorination was greatest with 16α-fluorine substitution, presumably due to an active 16α-hydroxylase activity. Three of the most promising compounds, 16β-[18F]fluoro-5α-dihydrotestosterone, 16β-[^18^F]fluoro-mibolerone, and 20-[^18^F]fluoro-mibolerone were further investigated in a baboon, which is a nonhuman primate model [27]. All three compounds had shown nearly equivalent AR binding affinity and AR-specific uptake in the prostate of androgen-suppressed rats [26], but they had very different affinities for the serum steroid-carrier protein, sex steroid hormone-binding globulin (SHBG). The data are shown in Table 1. FDHT clearly provided better and more selective uptake than the other two in baboon prostates [11]. This difference is due to the greater affinity that FDHT has for the blood steroid-carrier protein, SHBG, than the mibolerone (Table 1).

Thus, FDHT was chosen as a compound for further studies in humans. The in vivo known routes of metabolism of different androgens are mediated by 3α-hydroxysteroid dehydrogenases (3α-re HSDs) and 5α-reductase (5α-R). FDHT demonstrates 10-fold affinity as compared to testosterone for AR. Both these enzymatic pathways are used for the design of androgen-related medication. However, FDHT is not affected by these enzymatic activities.

**Figure 3:**
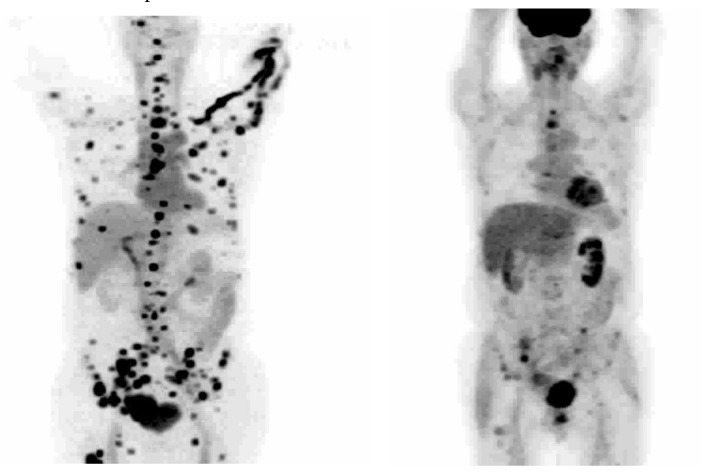
[^18^F]FDHT (**left**) and [^18^F]FDG (**right**) PET of patient with prostate cancer, demonstrating major differences in the uptakes. This cancer is AR-positive but FDG-avid only to a limited extent. These pictures are provided by Steven M. Larson (MSKCC, New York, NY, USA).

**Figure 4:**
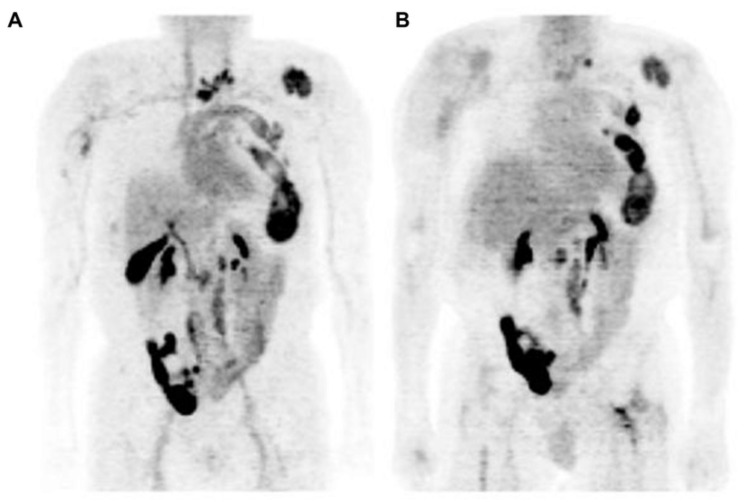
Comparison of [^18^F]FDHT (**left** (**A**)) and [^18^F]FDG (**right** (**B**)) PET in maximum-intensity projection (MIP) views with widespread bone metastases involving cervical spine, left ribs, and left para-aortic lymph nodes: physiological urinary activity in a right lower abdominal quadrant urinary diversion is also seen [28]. Note: [28] © by the Society of Nuclear Medicine and Molecular Imaging, Inc. (Reston, VA, USA).

16β-^18^F-Fluoro-5α-dihydrotestosterone ([^18^F]FDHT) is the radiolabeled analogue of dihydrotestosterone that directly binds to the AR. It allows in vivo visualization and quantification of AR expression. The first clinical study on the feasibility of AR imaging using PET and the binding selectivity of [^18^F]FDHT to AR in patients with prostate cancer was published in 2004 by Larson et al. [28]. Next year, the results of this study were supported by a study from Dehdashti et al. [14]. Both studies concluded that [^18^F]FDHT uptake is a receptor-mediated process. Dehdashti et al. reported the sensitivity and lesion detection rates of [^18^F]FDHT PET/CT to be 63% and 86%, respectively. The positive [^18^F]FDHT PET/CT results correlated with higher PSA level [14]. This one, as well as other studies [29,30,31], showed that [^18^F]FDHT PET/CT may have a more significant role in the management and prognostication of advanced prostate cancer rather than initial staging. The sensitivity of [^18^F]FDHT PET/CT was reported to be inferior to that of [^18^F]FDG PET/CT [28]. The authors of this study compared the [^18^F]FDHT and [^18^F]FDG PET/CT results from 59 lesions in seven patients with CRPC. Of them, 97% of these lesions were seen on [^18^F]FDG, while 78% were seen on [^18^F]FDHT PET/CT. Examples of the ^18^F]FDHT PET and [^18^F]FDG PET studies are shown in Figure 3 and Figure 4. In Figure 3, there are multiple FDHT lesions and only a few FDG lesions, whereas, in Figure 4, both FDHT and FDG demonstrate multiple weak lesions.

In a retrospective study looking for an association between imaging findings and overall survival in 39 patients with CRPC, the intensity of the [^18^F]FDHT uptake (defined as the highest bone lesion SUVmax (maximal standard uptake value) in the patient) correlated inversely with the overall survival, and patients with higher [^18^F]FDHT uptakes had shorter overall survival [30]. This was not the case for a correlation between the overall survival and [^18^F]FDG uptake.

The [^18^F]FDHT tumor uptake as well as the metabolism of the radiopharmaceutical is rapid, with an 80% conversion to radiolabeled metabolites that circulated while bound to plasma proteins (within 10 min). Because [^18^F]FDHT is rapidly metabolized in humans and excreted via the kidneys into urine, it may compromise the detection of tumor lesions close to the prostate. Table 2 demonstrates the clinical studies from the literature [14,28,30,32,33,34,35].

### 2.2. [^18^F]FDHT PET Analysis

The signal in [^18^F]FDHT is rather low when compared to many other tumor-targeting PET radiopharmaceuticals. Therefore, it is important to analyze how count statistics and reconstruction protocols affect its accuracy, repeatability, and lesion detectability. A study population of 14 metastatic mCRPC patients with a total of 336 [^18^F]FDHT-positive lesion scans were studied using PET/CT. The PET/CT were analyzed using four different validated reconstruction protocols demonstrating that reproducible scans and reliable images for detection will be obtained if the acquisition time is sufficient. More importantly, qualitative information was not essentially lost in this study. For example, the reduction in the acquisition time from 3 min to 1.5 min per bed position resulted in a repeatability of SUV values remaining at ≤30%, which is generally acceptable for response monitoring purposes [36]. In a multicenter study, healthy tissues with limited uptake variability were identified. The liver is the best reference organ to interpret the [^18^F]FDHT uptake [37].

The pharmacodynamic behavior is an essential factor to understand quantification prerequisites for noninvasive PET methods. The [^18^F]FDHT uptake was best described using an irreversible two-tissue-compartment model with a blood volume parameter (*Ki* = *k*_1_ × *k*_3_/(*k*_2_ + *k*_3_)) in mCRPC patients [38]. This was found in a study of 87 lesions by using dynamic [^18^F]FDHT PET/CT scans in 14 patients with venous blood sampling and in 6 patients with arterial blood sampling and dynamic ^15^O-H_2_O PET scans [38].

### 2.3. [^18^F]FDHT PET Prostate Cancer Imaging in Clinical Trial Design

[^18^F]FDHT PET imaging could be useful to assess AR signaling in clinical trials. We know from nonclinical studies that the phosphatidylinositol 3-kinase/Akt/mammalian target of the rapamycin (mTOR) pathway—PI3K pathway—activation is associated with repressed AR signaling. This may explain why the mCRPR phenotype can be observed in these PCa patients. Therefore, mTOR inhibition was tested in mCRPR patients [39]. Unfortunately, their phase I results showed limited antitumor activity, which is postulated to be due to the release of negative feedback on the PI3K pathway, and the toxicity was greater than anticipated. The trial was stopped because alternative AR/PI3K-directed combinatorial therapies performed better [39]. The effect was demonstrated using [^18^F]FDHT PET/CT imaging. In this phase I study, patients with mCRPR received 6 mg/kg cixutumumab (inhibits both the ERK-MAPK as well as the PI3K-AKT-mTOR pathway) and 25 mg temsirolimus intravenously each week [39]. The investigators measured the circulating tumor cells, [^18^F]FDG PET/CT, [^18^F]FDHT PET/CT, and performed tumor biopsies to understand the phenomena [39]. One case from this study demonstrated reciprocal feedback regulation of the PI3K and AR pathways providing an explanation for the paradoxical increase in both the [^18^F]FDHT PET uptake and in PSA response to mTOR-inhibitor temsirolimus, followed by a decline when the drug was subsequently stopped. Temsirolimus relieved the mTOR-mediated negative feedback on human epidermal growth factor receptor (hEGF) HER2 and HER3 signaling, which is upstream of AR [39]. The AR was activated and both PSA and [^18^F]FDHT SUV increased in this patient. By stopping temsirolimus, both the PSA and [^18^F]FDHT SUV decreased, because the restoration of the PI3K pathway’s negative feedback on EGF signaling. The knowledge of the presence of AR in combination with glycolytic activity assessed using FDG PET could be used as part of exclusion criteria, because it is known that the presence of glycolytic activity worsens to a small extent the prognosis of PCa [34].

### 2.4. New Targets for AR Imaging in Patients with Prostate Cancer

There are multiple nonsteroid ligands for AR that are developed for various endocrine therapeutic purposes, such as antiandrogens to treat prostate cancer (enzalutamide, apalutamide, darolutamide, and earlier flutamide and bicalutamide) and selective AR modulators (SARMs) for treating hypogonadal conditions in men. Because some of these have high AR binding affinity and are small and relatively polar compounds, they have been radiolabeled either using fluorine-18 (^18^F) or bromine-76 (^76^Br). Unfortunately, flutamide and bicalutamide were not targeting AR in the prostate in nonclinical models [40,41,42].

Enzalutamide is an AR signaling inhibitor that is currently used in different stages of prostate cancer. The mechanism of action for enzalutamide is threefold: (1) it is a potent, competitive binder of androgens at the level of the AR; (2) it prevents the translocation of the AR from the cytoplasm to the nucleus; and (3) in the nucleus, it inhibits AR binding to chromosomal DNA, which prevents the further transcription of tumor genes. Enzalutamide and its primary metabolite N-desmethylenzalutamide have an AR affinity comparable to that of FDHT but they are excreted mainly via the hepatic route. A lipophilic tracer could be a better candidate than a hydrophilic tracer (excretion via the nephrourinary tract). Radiolabeled enzalutamide could thus be a suitable PET radiopharmaceutical for AR imaging. In vivo PET and biodistribution studies on male mice bearing an LnCaP (AR+) xenograft showed an approximately three-times-higher tumor uptake for ^18^F-enzalutamide than for [^18^F]FDHT [43]. Sixty minutes after tracer injection, 93% of the ^18^F-enzalutamide in plasma was still intact when compared with only 3% of the [^18^F]FDHT. In a nonclinical study, ^18^F-enzalutamide showed a higher tumor uptake and better metabolic stability than [^18^F]FDHT [43] and thus seems to have more favorable properties for the imaging of AR using PET. This should be confirmed in other oncologic animal models and in patients.

## 3. [^18^F]FDHT PET in Patients with Breast Cancer

**Figure 5:**
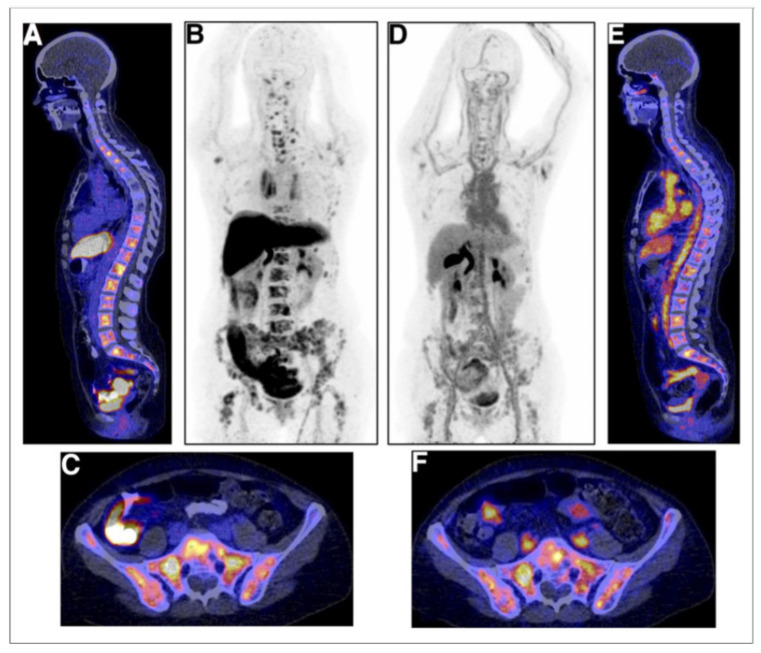
Examples of [^18^F]FES (**A**–**C**) and [^18^F]FDHT (**D**–**F**) studies in same breast cancer patient with multiple bone metastases. (**A**) [^18^F]FES PET/CT scan with physiological uptake in liver, small intestine, and urinary tract and pathological uptake in multiple vertebra. (**B**) [^18^F]FES PET MIP allow for visualization of [^18^F]FES biodistribution. (**C**) [^18^F]FES PET/CT with physiologic uptake in small intestine and pathological uptake throughout pelvic bones. (**D**) [^18^F]FDHT PET MIP, with physiologic uptake in heart and excretion through liver and urinary tract. (**E**) [^18^F]FDHT PET/CT with pathologic uptake in multiple vertebrae. (**F**) [^18^F]FES PET/CT with physiologic uptake in large vessels and small intestines and pathologic uptake throughout pelvic bones [47]. Note: [47] © by the Society of Nuclear Medicine and Molecular Imaging, Inc.

In general, ARs are not routinely measured in patients with breast cancer, despite being present in 70–80% of them. Because only estrogen-receptor-positive (ER+) patients benefit from antiestrogen therapy, and as ER patients are functionally and structurally comparable with AR, response to AR-targeting drugs may rely on AR expression as well [44]. [^18^F]FDHT PET can be used for assessing AR expression in breast cancer and ^18^F-fluoroestradiol ([^18^F]FES) PET for visualizing ER expression in tumor lesions [45]. Molecular imaging offers the possibility to noninvasively determine the presence of relevant drug targets in all sites of metastatic spread throughout the body [46]. In a study of Venema et al. [46], the correlation (R^2^) between semiquantitative AR expression and [^18^F]FDHT uptake was 0.47 (*p* < 0.01) and between semiquantitative ER expression and [^18^F]FES uptake was 0.78 (*p* < 0.01). They found an optimal cutoff for AR-positive lesions was an SUVmax of 1.94 for [^18^F]FDHT PET, resulting in a sensitivity of 91% and a specificity of 100%; the optimal cutoff was an SUVmax of 1.54 for [^18^F]FES PET, resulting in a sensitivity and specificity of 100% for ER. The tumor AR and ER expressions were measured immunohistochemically from biopsies within 8 weeks of the PET acquisition. The authors emphasize that these results show the potential use of AR and ER imaging for receptor status assessment, particularly in respect to biopsy sampling errors and heterogeneous AR and/or ER expression in breast cancer metastases [46]. A patient imaged both using FES and FDHT has been shown in Figure 5, and her lesions can be better visualized using FES compared to FDHT. Molecular imaging could be a promising tool in patient selection for clinical trials with AR antagonist treatment protocols. AR-targeted therapy has not been approved as a standard treatment regimen, but there are clinical trials with preliminary acceptable results, showing stable posttreatment disease in one-third of metastatic breast cancer patients. Many studies of AR-targeted therapy and a combination of AR- and ER-targeted therapies are ongoing, allowing for better perspective for patients with AR- and ER-positive metastatic breast cancer. Having in mind that hormone receptor conversion occurrence is common during the disease development while both receptor-positive and receptor-negative lesions are present, pretreatment ER and AR assessment is of great importance [44,45,47].

[^18^F]FDHT and [^18^F]FES PET could be used instead of metastasis biopsy when the lesions are not easily accessible. Additionally, [^18^F]FDHT and [^18^F]FES PET are very important for the assessment of treatment effectiveness, avoiding the suboptimal treatment when the receptors are changing in the process of metastatic disease development [45]. In a recent study, eleven postmenopausal women with ER metastatic breast cancer underwent [^18^F]FDHT PET/CT at baseline and at 6 and 12 weeks after starting selective AR modulation (SARM) therapy [48]. The median baseline [^18^F]FDHT SUVmax was 4.1 (1.4–5.9) for AR-positive tumors versus 2.3 (1.5–3.2) for AR-negative tumors (*p* < 0.22). Seven participants with clinical benefit at week 12 tended to have larger declines in [^18^F]FDHT uptake than those with progressive disease both at week 6 and week 12 after starting SARM [46]. For the assessment of the therapy response, surrogate markers to evaluate the presence of metastatic breast cancer cells are needed. This study on [^18^F]FDHT and earlier studies on FES in AR [48] and ER imaging [45,47] demonstrate that both FES and [^18^F]FDHT could act as imaging biomarkers for evaluating the response of metastatic breast cancer.

The [^18^F]FDHT uptake could be used to follow up patients with AR-positive metastatic breast cancer who had bicalutamide-induced reduction [49]. This observation was based on 349 lesions in 17 patients. However, this change could not predict bicalutamide response.

The SUV [^18^F]FDHT values are low in patients with breast cancer. In a study of Mammatas et al., 120 lesions were identified in 10 ER-positive metastatic breast cancer patients with either conventional imaging (bone scan or lesions larger than 1 cm on high-resolution CT, *n* = 69) or with [^18^F]FES and [^18^F]FDHT PET (*n* = 51) [50]. There was a high interobserver agreement in both the visual and quantitative evaluation of [^18^F]FES PET uptake supporting the use of [^18^F]FES PET in clinical practice. In contrast, the visual agreement for [^18^F]FDHT uptake was relatively low due to there being low tumor-background ratios, whereas the quantitative agreement was good. This study underscores the relevance of performing quantitative analysis of [^18^F]FDHT PET in breast cancer [50].

Table 3 summarizes the clinical studies from the literature [46,48,49].

## 4. [^18^F]FDHT PET in Patients with Other Cancers

Androgens can act through AR in the brain. [^18^F]FDHT PET has been used to image AR expression in the brain in an animal experiment where rats were either orchiectomized to inhibit endogenous androgen production or underwent sham surgery [51]. Fifteen days after surgery, a 90 min dynamic [^18^F]FDHT PET with arterial blood sampling was performed. Additionally, in a group of orchiectomized rats, 1 mg/kg dihydrotestosterone was co-injected with the tracer in order to saturate the AR. PET imaging and biodistribution studies showed low [^18^F]FDHT uptake in all brain regions, except in the pituitary gland. The [^18^F]FDHT PET uptake in the surrounding cranial bones was high and increased over time. The [^18^F]FDHT was rapidly metabolized in rats, and was significantly faster in orchiectomized rats than in the sham-orchiectomized rats. The [^18^F]FDHT uptake in the brain could not be blocked by endogenous androgens or administration of dihydrotestosterone [51].

All the results of this study indicate that the imaging of the AR availability in rat brains using [^18^F]FDHT PET is not feasible. The low AR expression in the brain, the rapid metabolism of [^18^F]FDHT in rats, and the poor brain penetration of the tracer likely contributed to the poor outcome of [^18^F]FDHT PET [51].

Despite the poor results in the AR targeting in the brain, [^18^F]FDHT PET may be used for glioma imaging. AR is overexpressed in 56% of glioblastoma multiforme (GBM) specimens and AR antagonists induced dose-dependent death in several GBM cell lines and significantly reduced tumor growth and prolonged the lifespan of mice implanted with human GBM. Twelve patients with suspected high-grade glioma underwent routine diagnostic protocols and additional dynamic and static imaging using [^18^F]FDHT PET/CT [52]. Visual and quantitative analyses of [^18^F]FDHT kinetics in the tumor and normal brain were performed. The SUVmean and SUVmax were determined in selected VOIs (volumes of interest) before surgery or biopsy. AR protein was analyzed in the tumor samples using Western blot [52].

In 6 out of the 12 patients, the [^18^F]FDHT uptake was significantly higher in the tumor when compared to the normal brain. The AR protein expression was also increased within the tumors. The tumor-to-normal brain SUVmean uptake ratio correlated positively with the AR protein expression, and this correlation was statistically significant (Pearson’s correlation coefficient r = 0.84; *p* < 0.002) [52]. Orevi et al., also presented two patients imaged using [^18^F]FDHT; one patient had a high glioma-associated tumor uptake and another patient with gliomatosis cerebri did not show any tumor uptake [52]. The dynamic imaging could confirm these differences with the time–activity curves of the SUVmax corresponding to the surgically extirpated areas and normal brain [52].

## 5. Conclusions

[^18^F]FDHT PET potentially provides a noninvasive method for the assessment of AR expression in patients with mCRPC. Keeping in mind that, during the disease development, hormone receptor conversion occurrence is common while both AR-positive and AR-negative lesions exist, receptor status assessment is of great importance in order to avoid biopsy, especially when the lesions are not easily accessible. This relatively new oncological tracer could be a promising candidate in PET patient selection for AR antagonist treatments. Despite the above-mentioned facts, the optimal use of [^18^F]FDHT PET in the clinical work with prostate cancer patients has not been clarified. [^18^F]FDHT PET has not yet entered routine clinical use, so further investigations are needed.

[^18^F]FDHT PET demonstrates relatively low tumor–background ratios when compared to [^18^F]FES PET, but the quantitative agreement was good. This highlights the relevance of quantitative analyzing [^18^F]FDHT PET in the response evaluation to the endocrine therapy of breast cancer. Despite there being limited statistics, the visual information of the presence of AR is crucial in selecting a therapy regimen.

## Figures and Tables

**Table 1:** Three androgen ligands evaluated in nonhuman primates. In androgen-suppressed adult male rats, %ID/g is at 1 h in the prostate (ventral), prostate/muscle ratio is at 4 h. Relative binding affinities (RBA) are relative to standards: for AR, methyltrienolone = 100; and for sex hormone-binding globulin (SHBG), estradiol = 1. For human applications, regulatory requirements included studies in two animal species, here in baboon and rat. Data modified from [22].

Compound	Prostate Uptake (%ID/g)	Prostate/Muscle Ratio	Relative Biologic Affinity to AR ^1^	Relative Biologic Affinity to SHBG ^2^
20-fluoromibolerone	0.97	5.5	53	4
16β-fluoromibolerone	0.67	3.8	31	1.3
16β-Fluoro-5α-dihydrotestosterone (FDHT)	0.57	5.4	43	385

^1^ AR =androgen receptor, RBA for methyltrienolone = 100; and ^2^ SHBG, RBA for estradiol = 1.

**Table 2:** Clinical studies with [^18^F]FDHT in patients with prostate cancer presented in the literature.

First Author, Year	No. of Patients with Prostate Cancer	Objective of the Study	[^18^F]FDHT PET	SUV_max_	Conclusions
**Larson 2004** [28]	7	To asses AR status		Average SUV(max) = 5.28	[^18^F]FDHT PET/CT localizes prostate cancer
**Zanzonico 2004** [32]	7	To derive estimates of normal-tissue-absorbed doses for [^18^F]FDHT			331 MBq (5 cGy/[0.0151 cGy/MBq]) is recommended for [^18^F]FDHT
**Dehdashti 2005** [14]	20	To evaluate the feasibility of [^18^F]FDHT PET/CT and to assess the binding selectivity of [^18^F]FDHT to AR			[^18^F]FDHT uptake is a receptor-mediated process. Positive PET studies were associated with higher PSA levels and thus, presumably, with greater tumor burden
**Fox 2011** [33]	20	Correlation between imaging signals of [^18^F]FDG and [^18^F]FDHT by readers	[^18^F]FDHT [^18^F]FDG	1.8–2.6 for the response assessment	99% concordance of identifying [^18^F]FDG and [^18^F]FDHT-negative sites, and positive-site agreement was 83% for [^18^F]FDG and 85% for [^18^F]FDHT. New method for response assessment
**Vargas 2014** [30]	38	To determine associations between [^18^F]FDHT PET/CT and overall survival			Patients with higher SUVmax on [^18^F]FDHT PET/CT had significantly shorter overall survival
**Fox 2018** [34]	133	To determine combined value of [^18^F]FDG and [^18^F]FDHT as prognosticator	[^18^F]FDHT [^18^F]FDG	Bone 5.5, lymph nodes 6.4, prostate 7.6	Three phenotypes were AR_1_Glyc_1_, AR_1_Glyc_0_, and AR_0_Glyc_1_ were identified. If AR is negative and FDG positive, it has a negative impact on survival
**Al Jalali 2023** [35]	10	Correlation between imaging signals of [^68^Ga]PSMA and [^18^F]FDHT	[^18^F]FDHT PET/MRI [^68^Ga]PSMA		Tumor detection rate of the [^68^Ga]PSMA was 90%, but only 40% for the [^18^F]FDHT PET/CT

**Table 3:** Clinical studies with [^18^F]FDHT in patients with breast cancer presented in the literature.

No of Pts; Sex; Histology	ER/AR Status Primary Tumor	FDHT/FU	SUV_max_	Response/Comment [% FDHT Change]	First Author Year
	Metastases				
11 female/2 male	13 ER+, 13AR+ 11 R+, 11AR+, 2AR−, 2ER− (10 AR+/ER+)	Sensitivity 91% Specificity 100%	Cut off for AR+ 1.94	Hormone receptor conversion in 23% metastases	Venema 2017 [46]
21 female 15 ductal, 6 lobular	14 ER+, 19 AR+, 5ER−	BL, 4–6 wk	17 pts w 349 lesions, decrease in SUV from 1.3 to 0.7 per patient and lesion	Bicalutamide response per patient −45%, per lesion −39%. −30% (nr) vs. −53% (nr) 341 of 515 lesions at BL	Boers 2021 [49]
	10 ER+, 15 AR+				
11 female 9 ductal, 2 lobular	11 ER+, 7AR+, 2AR−	BL, 6 wk, 12 wk	AR+ 4.1 AR− 2.3	SARM response 6 wk: −26.8 (r) vs. −3.7 (nr) 12 wk: −35.7 (r) vs. 20.1 (nr) 40 lesions	Jacene 2022 [48]

BL = baseline; r = responders, and nr = non-responders.

## Data Availability

Not applicable.

## Abbreviations

ACTH = adrenocorticotropic hormone; AR = androgen receptor; BL = baseline; CRH = corticotropin-releasing hormone; CT = computed tomography; DES = diethylstilbesterol; DHEA = dehydro-epiandrosterone; DHT = 5α-dihydrotesterone; ER = estrogen receptor; C = 2-fluorodeoxyglucose; FDHT fluoro-5α-dihydrotestosterone; [^18^F]FDHT = 16β-^18^F-Fluoro-5α-dihydrotestosterone; FES = 16α-[^18^F]fluoroestradiol; FFNP = 21-[^18^F]-fluoro-furanyl-nor-progesterone; FU = follow up; GBM = glioblastoma multiforme; Glyc = glycolysis measured by FDG; ID = injected dose; LH = luteinizing hormone; LHRH = luteinizing hormone-releasing hormone; mCRPC = metastatic castration-resistant prostate cancer; PCa = prostate cancer; PET = positron emission tomography; PSA = prostate-specific antigen; RBA = relative binding affinity; SARM = selective androgen receptor modulation; SHBG = sex steroid hormone binding globulin; SUV = standard uptake value; and VOI = volume of interest.
